# Effects of repeated sprint exercise on brachial artery shear rate patterns in healthy adults

**DOI:** 10.14814/phy2.70523

**Published:** 2025-08-26

**Authors:** Matthew A. Chatlaong, Lori M. Keys, Matthew B. Jessee

**Affiliations:** ^1^ Exercise Science—School of Human Services University of Cincinnati Cincinnati Ohio USA; ^2^ Applied Human Health and Physical Function Laboratory, Department of Health, Exercise Science, and Recreation Management University of Mississippi University Mississippi USA

**Keywords:** cardiovascular responses, endothelium, interval, nitric oxide, vascular function, vasoconstriction

## Abstract

Endothelial cells are influenced by mechanical shear forces from blood flow, which increase considerably during exercise. Exercise‐induced shear stimuli depend on several factors, including intensity and modality; though little is known about the effects of sprint exercise. This study investigated the effects of maximal sprint exercise on systemic shear patterns. Eighty‐four healthy participants completed 2 × 20 s maximal cycling sprints (5‐min rest between) or 10 min of moderate intensity (64%–76% HR_max_) cycling (MOD). Ultrasound recordings of the brachial artery were taken at baseline and minutes 1 (M1), 3 (M3), and 5 (M5) of recovery after sprint 1 (S1), sprint 2 (S2), and MOD, and again 10‐ and 15‐min post‐exercise. Bayesian rmANOVA was used to compare shear rate and diameter over time. Average and antegrade shear rate increased after each sprint and were not different between sprints at M1 and M3 (all BF_10_ ≤ 0.545), but antegrade was higher for S2 at M5 (BF_10_ = 40.417). Retrograde shear developed progressively throughout. Elevated antegrade and retrograde shear responses were observed through 15 min in sprints but returned toward baseline by 10–15 min in MOD. Lasting effects of sprint exercise on non‐local (systemic) shear patterns could promote vascular remodeling. Future training intervention studies should investigate potential long‐term benefits.

## 1 INTRODUCTION

The progression of certain cardiovascular disease states is mediated by declining vascular function (Olsen et al., 2016). For example, endothelial dysfunction has been linked to the development of hypertension (Kaess et al., 2012), carotid intima‐media thickening (Halcox et al., 2009), atherosclerotic plaque (Gimbrone & García‐Cardeña, 2016; Matsuzawa & Lerman, 2014; Vanhoutte et al., 2017), and future cardiovascular events (Shechter et al., 2014). Reduced nitric oxide (NO) bioavailability is a central feature of endothelial dysfunction (Janaszak‐Jasiecka et al., 2023). Poor NO bioavailability compromises endothelial‐driven vasodilatory effects and protection of the vessel wall from platelet adhesion (Förstermann et al., 2017). Thus, preserving endothelial function is essential for vascular health.

Interestingly, endothelial cell function is influenced by wall shear stress from frictional forces generated by blood flowing through the vessel lumen (Green et al., 2017; Vanhoutte et al., 2017). Acute exposure to high blood flow can increase NO production by endothelial nitric oxide synthase (eNOS) (Balligand et al., 2009; Vanhoutte et al., 2017), which may be a key mechanism underpinning the therapeutic effects of exercise on vascular function (Green et al., 2017). Chronically, these responses help regulate eNOS expression (Vanhoutte et al., 2017) and long‐term vessel remodeling (e.g., vessel enlargement) (Tronc et al., 1996). In vivo, blood flow is pulsatile, and shear stress can include both antegrade (forward) and retrograde (backward) components. Importantly, endothelial cells appear sensitive to the different wall shear stresses imparted by antegrade and retrograde flow direction (Lu & Kassab, 2004; Wang et al., 2013). Patterns of antegrade and retrograde shear rate can be assessed noninvasively using Doppler ultrasound in conduit arteries (Birk et al., 2012; Tinken et al., 2010; Walsh et al., 2017), and previous studies have shown that patterns differ across exercise modalities and intensities (Montalvo et al., 2022). Since wall shear stress is a key hemodynamic stimulus for exercise‐induced remodeling, investigating the magnitude and time course of distinct shear patterns delivers insight into the therapeutic potential of a modality.

Recently, there has been growing interest in brief bouts of intense exercise and whether they could improve vascular function (Islam et al., 2022); though there is a sizeable knowledge gap around hemodynamic responses and vascular effects. For instance, a line of research investigating dose‐responses to sprint interval training has demonstrated that a 2 × 20‐s cycling sprint intervention can improve cardiorespiratory fitness (Cuddy et al., 2019; Metcalfe et al., 2016, 2020); though the implications on the vasculature remain unclear. The current body of literature investigating the effects of sprint exercise training on vascular function in healthy adults consists mainly of a few small studies, with some (Cocks et al., 2016; Petrick et al., 2021; Rakobowchuk et al., 2008) but not all (Shenouda et al., 2017) suggesting potential benefits. More work in this area is warranted.

There are previous investigations of acute hemodynamic responses to interval training modalities (Baughman & Sawyer, 2024; Lyall et al., 2019; Ogoh et al., 2021), but to our knowledge, there are very few reports of shear patterns in response to supramaximal sprinting. DeBlois et al. (2020) assessed shear patterns in the superficial femoral artery following a maximal 30‐s sprint on a cycle ergometer, showing increased antegrade and decreased retrograde shear rate 2 min post exercise. Importantly, several key unknowns remain, including what occurs in the systemic non‐local vasculature (e.g., in the arm), which is central for overall cardiovascular health. During continuous submaximal cycling, both antegrade and retrograde shear exhibit intensity‐dependent increases in non‐local vessels (i.e., brachial artery) (Baughman & Sawyer, 2024; Coovert et al., 2018; Gurovich et al., 2021; Tanaka et al., 2006). These responses have not been previously investigated during sprinting, which could elicit greater sympathetic outflow given rapid rest‐to‐maximal intensity transitions. Moreover, from the current literature it is unclear whether multiple maximal sprint efforts affect or augment these patterns, and to what extent effects persist throughout a longer post‐exercise recovery period. Addressing these questions is needed for a more comprehensive understanding of the hemodynamic stimulus from sprint exercise.

Accordingly, our aim was to investigate the effects of 2 × 20 s maximal cycling sprints on shear responses in the brachial artery, and for additional context beyond that provided in earlier studies, comparisons were also made with continuous moderate‐intensity exercise. Our hypotheses were as follows: (1) there would be marked changes in shear rates remaining for up to 15 min following sprint exercise, but that responses would return to baseline within 5–10 min after moderate intensity exercise; (2) peak shear rate responses observed immediately after each sprint would be greater than those observed in moderate intensity exercise, and (3) shear rates would be similar following each sprint repetition, i.e., that multiple efforts would not augment responses.

## 2 MATERIALS AND METHODS

### 2.1 Participants

A total of 91 recreationally active healthy female and male adults participated in this study, sampled from the surrounding university community. Descriptive characteristics are shown in Table 1. Exclusion criteria were regular use of nicotine products in the prior 6 months, use of medication known to affect heart rate or blood pressure, or any orthopedic injury that would prevent exercise on a cycle ergometer. All participants were screened via the Physical Activity Readiness Questionnaire (PAR‐Q+, 2023) and deemed ready to participate in physical activity. Prior to visiting the laboratory, all participants were asked to avoid exercise for 24 h, alcohol for 24 h, and food for 2 h. The study protocol received ethical approval by the University of Mississippi Institutional Review Board (22‐042 & 23‐017), and participants gave written consent to take part in the study after being informed of all procedures, risks, and study objectives.

**TABLE 1:** Descriptive data for participants in sprint and moderate intensity samples.

Characteristic	Sprints		Moderate	
	Female (*n* = 21)	Male (*n* = 21)	Female (*n* = 21)	Male (*n* = 21)
Age (y)	25.2 (7.7)	26.0 (5.0)	22.6 (6.0)	25.3 (6.1)
Height (cm)	166.7 (6.2)	173.3 (6.9)	166.7 (8.3)	178.9 (8.0)
Weight (kg)	59.2 (8.5)	79.0 (15.4)	69.5 (14.7)	81.4 (14.1)

*Note*: Data are mean (*SD*) for participants retained in analysis.

### 2.2 Exercise protocols

**FIGURE 1:**
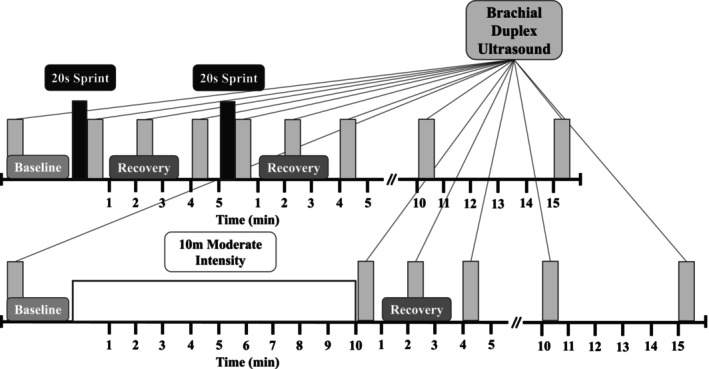
Visit timeline for sprint and moderate intensity bouts. Inset (top right) with connected lines denotes brachial artery ultrasound measures at each indicated time point.

Participants completed either sprint (*n* = 49) or continuous moderate exercise (*n* = 42). The sprint bout consisted of 2 × 20 s cycling sprints in a 10‐min period (Metcalfe et al., 2012, 2016, 2020; Songsorn et al., 2016) (Figure 1). An electronically braked cycle ergometer (Excalibur Sport 2006, Lode) and its Wingate software were used. Per manufacturer recommendations, ergometer resistance was set to 0.67 Nm/kg for females and 0.70 Nm/kg for males. Power output was recorded throughout each sprint effort using the ergometer software. For each sprint, participants began with pedals stationary (i.e., no pedaling occurred before resistance was added) and were instructed to sprint as hard and fast as possible for the full 20‐s period. Strong verbal encouragement was given throughout each effort. There was a 5‐min recovery period between each sprint (Figure 1). To compare between sprints and to assess responses to sprints alone (in the absence of continuous exercise), no active warm‐up or cool down was completed. Forty‐two of 49 participants from the sprint group were included in the final analysis, as four were unable to complete both sprints due to dizziness and/or nausea after the first sprint effort. Two participants developed similar symptoms during ultrasound measures following the second sprint and measures were not finished, and one participant exhibited very low brachial blood flow during heavy breathing and was removed from analysis, leaving 42 total participants after listwise deletion (Table 1).

Continuous moderate intensity exercise consisted of a 10‐min bout, chosen to match the length of the sprint bout (Figure 1), which has been recommended following previous investigations comparing shear stimuli across modalities (Montalvo et al., 2022). Participants cycled at a self‐selected cadence between 60–90 revolutions per minute while investigators adjusted workload to maintain heart rate (HR) between 64%–76% of age‐predicted maximum (ACSM, 2022), set by the equation from Gellish et al. (2007). HR was monitored throughout exercise sessions via a Bluetooth chest strap (H7, Polar). Forty‐two participants completed the moderate intensity cycling bout, and all were retained in the analysis (Table 1).

### 2.3 Ultrasound imaging

Throughout exercise bouts, continuous ultrasound video recordings of the brachial artery (HS60, Samsung) were taken in the right arm. Following internal pilot studies, it was deemed that high‐quality measures were unfeasible during sprint exercise, so they were taken at pre‐determined time points before and after each sprint. To control for differences due to body position, measurement technique was standardized at each measurement time point (Newcomer et al., 2008). Participants remained upright on the ergometer (no pedaling) with their back against a padded wall and right arm outstretched and supported on an elevated, padded surface. For the sprint bout, measurement time points were baseline, immediately following each sprint (denoted S1 and S2) in the first minute of recovery (M1), and again in minutes 3 (M3) and 5 (M5) of recovery (Figure 1). Additional measures were taken 10 and 15 min post exercise (10M and 15M) For the continuous moderate intensity bout, measurement time points were baseline and immediately following the 10 min of pedaling in min 1, 3, and 5 of recovery (Figure 1), and again 10 and 15 min post exercise (denoted M1, M3, M5, 10M, and 15M). No attempt was made to make measurements early in the continuous bout (e.g., to align with sprint 1) to avoid interrupting the continuous nature of the exercise and steady‐state cardiovascular response.

For all measures, a gel‐coated linear array probe (LA3–14 AD, Samsung) was held approximately 2–3 cm proximal to the antecubital fossa. Before exercise, the probe was positioned to make the artery visible across the entire screen; then the sample volume was adjusted to encompass the entire artery lumen. The probe angle and software settings were adjusted to maintain an insonation angle of <60 (Limberg et al., 2019). Probe location was marked using surgical tape placed on the skin, along the boundary of the probe, to ensure that the same location was imaged with repeated measurements.

At each measurement interval, duplex and B mode ultrasound were used sequentially (30 s duplex, then 5–10 s B mode) to record brachial artery blood velocity and diameter, respectively. Videos were analyzed offline using validated FloWaveUS software to obtain diameter and shear rate values (Coolbaugh et al., 2016). Mean, antegrade, and retrograde shear rates were analyzed for each measure. Multiple baseline measures were not taken; however, previous investigations from our laboratory have shown good to excellent test–retest reliability for brachial artery blood flow and shear rate (Stanford et al., 2022). Test–retest correlations calculated from similar measures at 10M and 15M in the current study were *r* = 0.96 for artery diameter and ranged from 0.72 to 0.80 for mean, antegrade, and retrograde shear rate.

### 2.4 Statistical analysis

Participant characteristics were compared between groups using Bayesian independent *t*‐tests. Peak and mean power output during each sprint were compared using Bayesian dependent *t*‐tests. Within each condition, the time course of exercise responses and return to baseline was assessed using a Bayesian repeated measures ANOVA approach with Bayesian paired *t*‐tests. To determine whether responses were augmented following the second sprint, values were compared at M1, M3, and M5 between S1 and S2. Peak and mean heart rate in the five minutes following each sprint were compared using Bayesian paired *t*‐tests. Responses to moderate intensity and sprint exercise were compared in a similar manner. Change scores from baseline (i.e., exercise induced changes) were calculated to account for subject‐level baseline variability and compared at M1, M3, M5, 10M, and 15M using Bayesian independent *t* tests. Peak and mean heart rate recorded throughout each exercise bout were compared using Bayesian independent *t*‐tests.

The Bayesian approach was used because it simultaneously compares the likelihood of the null hypothesis, that two means are the same, versus the alternative hypothesis that they are different (Wagenmakers et al., 2018). The likelihood of the null hypothesis is not assessed when using more traditional frequentist null hypothesis tests of difference (i.e., if *p* > 0.05, means are considered “not different”) (Wagenmakers et al., 2018). The Bayes factor (BF_10_) provides a likelihood ratio; for example, BF_10_ = 3 suggests that the alternative hypothesis is three times more likely than the null hypothesis. A classification scheme for BF_10_ was used (van Doorn et al., 2021). For those interested, we have also provided *p* values from analogous frequentist tests throughout, although it was decided a priori to base conclusions on the Bayesian analysis. Analyses were conducted using JASP (version 0.19.2) and the statsExpressions package in R software (Patil, 2021) using default JZS priors (*r* scale = 0.707) for all models. All data are presented as mean ± *SD* and mean [95% credible interval] unless otherwise noted.

## 3 RESULTS

### 3.1 Participant characteristics

Participant characteristics are shown in Table 1. There was no evidence of differences in age (BF_10_ = 0.433, *p* = 0.227), height (BF_10_ = 0.561, *p* = 0.154), or weight (BF_10_ = 0.977, *p* = 0.070) between sprint and moderate intensity groups.

### 3.2 Sprint power output

There was strong evidence that peak power output was higher during sprint 1 (933.2 ± 330.2 W) versus sprint 2 (879.2 ± 311.5 W, BF_10_ = 55.6, *p* < 0.001). There was also strong evidence that mean power output was higher during sprint 1 (577.3 ± 173.9 W) versus sprint 2 (543.6 ± 161.4 W, BF_10_ = 1121.3, *p* < 0.001).

### 3.3 Shear rate

#### 3.3.1 Mean shear rate

**FIGURE 2:**
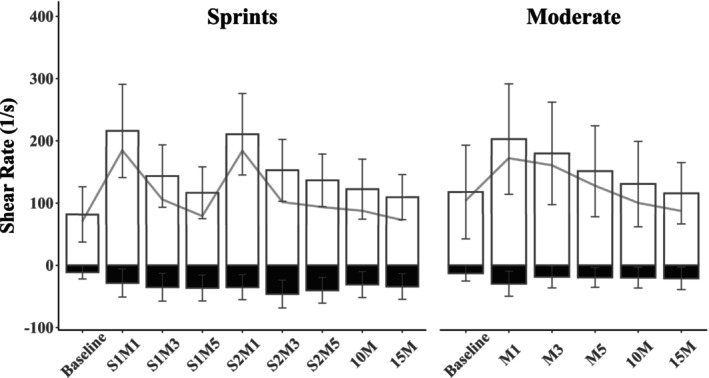
Brachial artery shear rate (1/s). Time point abbreviations denote the minute following the effort (M1‐M5) and sprint number (S1 or S2). White bars denote antegrade shear rate, and black bars show retrograde. The line connecting each bar denotes the mean shear rate. Error bars are the *SD* for antegrade and retrograde shear rates.

**FIGURE 3:**
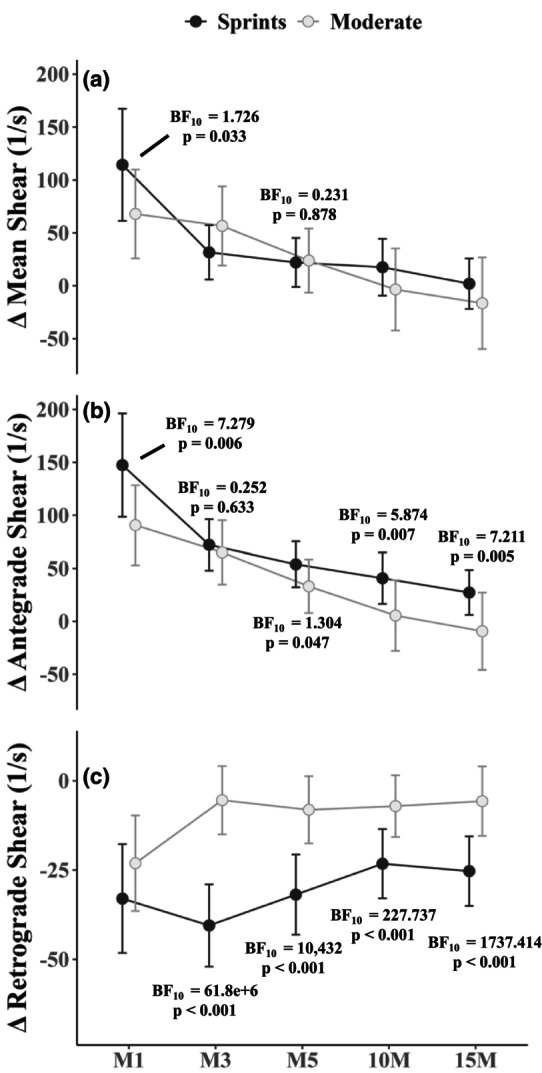
Changes in mean (a), antegrade (b), and retrograde (c) shear rate over time in each condition. Time point abbreviations denote the minute in recovery after each bout (M1 – 15M). Data are mean and *SD* (error bars) change scores from baseline.

Repeated measures ANOVA's indicated strong evidence of a change in mean shear rate (consisting of both antegrade and retrograde components) over time in both sprint (ANOVA BF_10_ = 8.75e+34, *p* < 0.001) and moderate intensity (ANOVA BF_10_ = 4.15e+10, *p* < 0.001) conditions (Figure 2). In the sprint bout, there was moderate evidence that increases observed at M1 and M3 following each sprint were similar (BF_10_ both ≤0.199, Table 2). At M5, however, there was moderate evidence that mean shear rate remained elevated after the second sprint (BF_10_ = 8.811, Table 2), while there was weak evidence of mean shear rate having returned toward baseline in the first (BF_10_ = 0.370, Table 2). There was moderate evidence that mean shear rate fully returned to baseline levels by 15M (BF_10_ = 0.175, Table 2). A similar trend was observed in moderate intensity with increased mean shear rate at M1, M3, and M5, though values returned to baseline by 10M (BF_10_ = 0.174, Table 3). Comparing sprint and moderate intensity responses, there was weak evidence in favor of a greater response in sprints at M1 (Figure 3a), but not at any other time point (BF_10_ all ≥0.231 and ≤0.881, *p* all ≥0.072, Figure 3a).

**TABLE 2:** Time course of changes in shear rate (1/s) throughout the sprint bouts.

Time 1	Time 2	Mean shear	BF_10_	*p*	Antegrade shear	BF_10_	*p*	Retrograde shear	BF_10_	*p*
BL	S1M1	112.23 (83.75, 139.49)	6.62e+07	<0.001	134.48 (112.01, 155.70)	7.44e+12	<0.001	−20.28 (−29.30, −11.47)	1190.506	<0.001
BL	S1M3	34.39 (21.57, 48.21)	6029.578	<0.001	61.33 (48.11, 75.48)	6.70e+08	<0.001	−26.22 (−32.65, −19.15)	2.20e+07	<0.001
BL	S1M5	8.30 (−4.93, 22.08)	0.370	0.200	32.99 (18.76, 48.07)	1088.972	<0.001	−26.77 (−32.89, −20.27)	2.95e+08	<0.001
BL	S2M1	111.06 (77.77, 143.84)	5.36e+05	<0.001	144.73 (113.83, 174.97)	1.72e+09	<0.001	−32.01 (−41.57, −22.28)	5.77e+05	<0.001
BL	S2M3	29.71 (13.57, 45.63)	85.388	<0.001	70.61 (54.82, 86.15)	1.23e+09	<0.001	−40.04 (−47.29, −32.42)	1.32e+11	<0.001
BL	S2M5	20.73 (6.02, 34.76)	8.811	0.004	52.66 (38.24, 66.05)	1.34e+07	<0.001	−31.21 (−38.51, −24.31)	3.26e+08	<0.001
BL	10M	16.33 (−0.20, 33.08)	1.191	0.043	39.18 (23.49, 54.57)	5505.312	<0.001	−22.61 (−28.87, −16.13)	5.29e+06	<0.001
BL	15M	1.91 (−12.19, 16.50)	0.175	0.788	26.00 (12.75, 39.39)	146.114	<0.001	−24.66 (−30.88, −18.28)	3.59e+07	<0.001
S1M1	S2M1	−0.48 (−21.71, 21.52)	0.169	0.963	10.47 (−11.07, 32.69)	0.270	0.321	−10.97 (−19.68, −2.15)	3.596	0.011
S1M3	S2M3	−4.12 (−18.50, 9.86)	0.199	0.557	8.66 (−2.81, 20.16)	0.545	0.117	−13.21 (−19.10, −7.47)	873.659	<0.001
S1M5	S2M5	11.99 (−0.49, 24.40)	1.023	0.052	18.04 (7.70, 28.30)	40.417	<0.001	−4.17 (−10.68, 1.86)	0.402	0.177
10M	15M	−14.61 (−25.71, −4.09)	4.692	0.008	−12.69 (−21.73, −4.10)	7.115	0.005	−1.94 (−6.85, 2.70)	0.230	0.419

*Note*: Time point abbreviations denote sprint number (S1 or S2) and minute following the sprint (M1‐M5). Values are posterior estimates of mean differences from Bayesian *t* tests, denoted as time in column 2 – column 1 (95% credible interval).

**TABLE 3:** Time course of changes in shear rate (1/s) throughout the moderate‐intensity bout.

Time 1	Time 2	Mean shear	BF_10_	*p*	Antegrade shear	BF_10_	*p*	Retrograde shear	BF_10_	*p*
BL	M1	65.03 (39.30, 91.02)	3663.464	<0.001	88.27 (64.82, 111.69)	8.97e+06	<0.001	−22.16 (−30.41, −13.75)	1.07e+04	<0.001
BL	M3	53.51 (31.30, 77.78)	1302.528	<0.001	62.74 (44.42, 82.45)	7.11e+05	<0.001	−5.10 (−10.67, 0.57)	0.776	0.073
BL	M5	22.22 (4.39, 40.95)	2.93	0.014	31.45 (15.99, 47.35)	204.95	<0.001	−7.63 (−13.30, −1.84)	4.908	0.007
BL	10M	−3.41 (−25.45, 20.11)	0.174	0.771	4.96 (−13.88, 25.33)	0.191	0.592	−6.67 (−11.79, −1.38)	3.681	0.011
BL	15M	−15.35 (−40.26, 10.05)	0.338	0.224	−8.60 (−29.54, 12.81)	0.229	0.415	−5.31 (−10.98, 0.42)	0.855	0.065
10M	15M	−12.24 (−27.38, 3.17)	0.539	0.117	−14.04 (−27.58, −0.16)	1.095	0.047	1.19 (−3.38 6.05)	0.193	0.582

*Note*: Time point abbreviations denote minute following exercise. Values are posterior estimates of mean differences from Bayesian *t*‐tests, denoted as time in column 2–column 1 (95% credible interval).

#### 3.3.2 Antegrade and retrograde shear rates

Repeated measures ANOVA's indicated strong evidence of changes in antegrade (ANOVA BF_10_ = 3.32e+52, *p* < 0.001) and retrograde (ANOVA BF_10_ = 3.59e+13, *p* < 0.001) shear rate over time for sprints (Figure 2). The same was observed for antegrade (ANOVA BF_10_ = 2.07e+19, *p* < 0.001) and retrograde (ANOVA BF_10_ = 7.26e+07, *p* < 0.001) in moderate intensity (Figure 2). In contrast to mean shear rate, however, neither antegrade nor retrograde shear rate returned to baseline after the sprints; that is, there was strong evidence that both remained elevated through 15M (BF_10_ all ≥146.114, Table 2). Comparing between S1 and S2, there was strong evidence that antegrade shear rate was augmented after S2 at M5 (BF_10_ = 40.417, Figure 2, Table 2), but not M1 and M3. Retrograde shear rate developed more in the second sprint, with moderate‐to‐strong evidence that it was greater following S2 at M1 and M3 (Figure 2, Table 2). While antegrade and retrograde shear rates increased from baseline in moderate intensity (Figure 2, Table 3), there was moderate evidence that antegrade shear rate returned toward baseline by 10M (BF_10_ = 0.191, Table 3). For retrograde shear rate, however, there was moderate evidence that it remained elevated through 10M (BF_10_ = 3.681, Table 3) and weak evidence of a return toward baseline by 15M (BF_10_ = 0.855, Table 3). Comparing sprint and moderate intensity responses, there was moderate evidence favoring greater antegrade responses in sprints at M1, 10M, and 15M, but not M3 (Figure 3b). There was weak evidence of greater antegrade responses in sprints at M5 (Figure 3b). Comparing retrograde shear rate between conditions, there was weak evidence of similar responses at M1 (BF_10_ = 0.546, *p* = 0.160), but strong evidence of greater retrograde shear rate in sprints at all other time points (Figure 3c).

### 3.4 Vascular diameter responses

**FIGURE 4:**
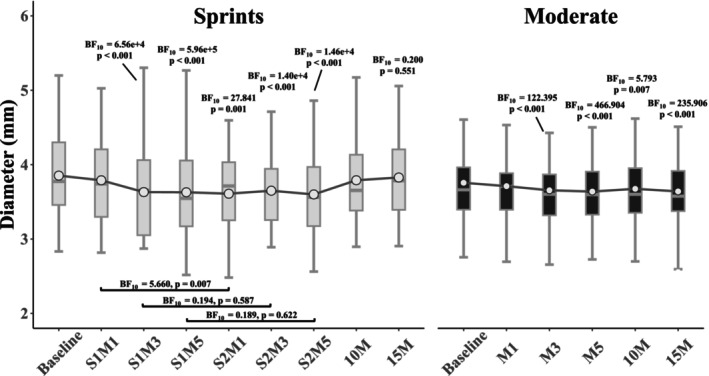
Brachial artery diameter (mm). Time point abbreviations denote the minute following the effort (M1‐M5) and sprint number (S1 or S2). White dots are means, connected by a line denoting the mean difference. Unless denoted by a black bar between time points, statistical comparisons denote time point versus baseline.

Repeated measures ANOVA's indicated strong evidence of a change in artery diameter over time in both sprint (ANOVA BF_10_ = 8.88e+12, *p* < 0.001) and moderate intensity (ANOVA BF_10_ = 428.048, *p* < 0.001) conditions (Figure 4). In both conditions, there was strong evidence that artery diameter decreased by M3 (S1M3 for sprints) (Figure 4). The decrease in artery diameter was not augmented by the second sprint, with moderate evidence that diameters were similar at M3 and M5 in both sprints (Figure 4). There was moderate evidence that artery diameter returned to baseline at 15M in sprints (Figure 4); however, there was strong evidence that it remained below baseline through 15M in moderate intensity (Figure 4). Post‐exercise vasodilatory responses, that is, an increase in artery diameter above baseline, were not observed in either condition. There was weak to moderate evidence favoring greater decreases in arterial diameter in M1–M5 following the sprint bout when compared to moderate intensity (BF_10_ all ≥ 2.652, Table 4). There was moderate evidence of responses being similar between conditions at 10M, but weak evidence of a difference due to arterial diameter remaining decreased at 15M in the moderate condition (BF_10_ = 1.666, Table 4).

**TABLE 4:** Comparison of change in diameter from baseline between sprints and moderate intensity at each post‐exercise measurement time point.

Comparison		Δ diameter (mm)	BF_10_	*p*
Sprints M1	Mod M1	0.175 (0.038, 0.323)	5.125	0.009
Sprints M3	Mod M3	0.091 (0.011, 0.174)	2.652	0.019
Sprints M5	Mod M5	0.105 (0.014, 0.204)	2.941	0.018
Sprints 10M	Mod 10M	−0.015 (−0.092, 0.063)	0.249	0.650
Sprints 15M	Mod 15M	−0.080 (−0.160, −0.001)	1.666	0.035

*Note*: Time point abbreviations denote minute following each bout (M1‐15M). Mod = moderate intensity bout. Values are posterior estimates of mean differences from Bayesian *t*‐tests, denoted as moderate‐sprints (95% credible interval). Positive values reflect a greater decrease in artery diameter for sprints.

### 3.5 Heart rate responses

**FIGURE 5:**
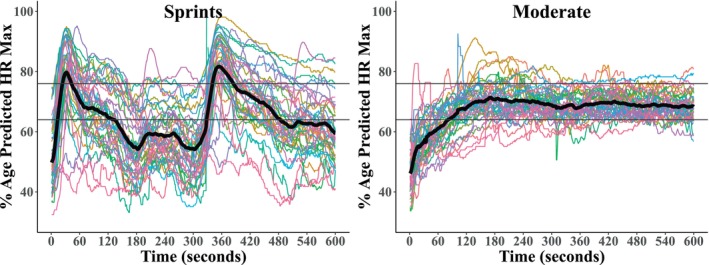
Heart rate throughout both bouts, expressed as a percentage of age predicted maximum heart rate. The thick black line shows the group average, and the other lines are individual tracings. The horizontal black lines denote target heart rate for moderate intensity.

Heart rate responses to both bouts are shown in Figure 5. Transient increases in heart rate were observed following each sprint, in comparison with overall steady‐state heart rate in the moderate‐intensity range during continuous cycling. Intermittent signal disruption in one recording for each group resulted in inclusion of *n* = 41 for both sprints and moderate bouts in analyses. During the sprint bout, there was strong evidence that peak heart rate was higher following sprint 2 (162.4 ± 18.2 bpm) versus sprint 1 (159.0 ± 17.5 bpm, BF_10_ = 17.026, *p* = 0.002). There was also strong evidence that mean heart rate over the 5 min following each sprint was also higher following sprint 2 (124.5 ± 17.8 bpm) versus sprint 1 (118.3 ± 16.8 bpm, BF_10_ = 3335.559, *p* < 0.001). While there was strong evidence of peak heart rate being higher in the sprint bout (163.4 ± 17.8 bpm) than moderate intensity (146.2 ± 9.4 bpm, BF_10_ = 27032.683, *p* < 0.001), there was weak evidence favoring higher mean heart rate throughout the moderate intensity bout (127.3 ± 6.2 bpm) compared to the sprints (121.7 ± 16.9 bpm, BF_10_ = 1.296, *p* = 0.050).

## 4 DISCUSSION

The aim of this study was to examine the effects of 2 × 20‐s maximal cycling sprints on systemic shear patterns. The findings clearly demonstrate robust increases in shear rate, marked vasomotor responses, and lasting effects on shear patterns throughout recovery. Previous investigations have reported increased antegrade and decreased retrograde shear rate in the leg after a 30‐s sprint (DeBlois et al., 2020); the current study extends these findings to responses in the non‐local vasculature for up to 15 min after sprint efforts with further comparison to moderate intensity exercise.

In agreement with our hypotheses, there was evidence that the highest observed antegrade, retrograde, and mean shear responses generated by sprint exercise were greater than those observed following moderate intensity (Figure 3). Higher peak heart rate values were also observed during sprinting (Figure 5). Decreased power output alongside increased heart rate in the second sprint indicated that sprint exercise was fatiguing – an effect we expected would impact post‐exercise shear responses. It was confirmed that at 10M and 15M, antegrade and retrograde shear rates remained elevated after sprinting, in contrast to the moderate condition where values returned toward baseline. This suggests possible ongoing stimuli for endothelial remodeling well after the cessation of exercise, and future work could incorporate additional follow‐up measures to determine how long effects last. These effects are particularly interesting in the context of prescribing brief exercise bouts to attenuate the negative impact of prolonged inactivity (Islam et al., 2022). Not without caution, however, the lasting changes in antegrade and retrograde shear patterns may cancel each other on average, as the overall mean shear rate returned to baseline by 15M following the sprints (Table 2). A further consideration is that some might interpret increasing retrograde shear as an adverse effect, since prolonged continuous flow reversal (Lu & Kassab, 2004) and low‐magnitude oscillatory shear (De Keulenaer et al., 1998; Tremblay et al., 2019) may negatively impact endothelial cell function. The effects of transient exercise‐induced increases in pulsatile retrograde shear as observed here remain unclear, especially with concurrently high antegrade shear yielding a robust net forward flow. Overall, increased retrograde shear is observed consistently during most exercise modalities (Coovert et al., 2018; Gurovich et al., 2021; Montalvo et al., 2022), and exercise training seems to improve endothelial function (Ashor et al., 2015; Khalafi et al., 2022; Tao et al., 2023).

These findings provide an important characterization of the hemodynamic stimulus to brief sprint interval exercise; though it should be considered that it is unclear whether this is sufficient to drive vascular remodeling. We would speculate that the responses observed in the current study would contribute to endothelial NO production and possibly vascular endothelial remodeling over time; although this remains to be confirmed experimentally via longitudinal exercise training studies. To our knowledge, there are only a few previous studies investigating the effects of sprint interval training on vascular outcomes in healthy adults, with some (Cocks et al., 2016; Petrick et al., 2021; Rakobowchuk et al., 2008) but not all (Shenouda et al., 2017) suggesting potential benefits. Shenouda et al. (2017) assessed several upper‐ and lower‐body vascular function outcomes following 12 weeks of training with 3 × 20 s sprints, compared to moderate intensity continuous training (45 min at 70% HR_max_) and non‐exercise control groups. Interestingly, with the exception of an improvement in allometrically scaled (but not unscaled) brachial flow‐mediated dilation following moderate intensity training, no changes in upper‐ or lower‐body vascular function outcomes were observed in either training group. Based on our results and other training studies (Cocks et al., 2016; Petrick et al., 2021; Rakobowchuk et al., 2008), we would contend that 45 min of moderate intensity exercise likely provides a powerful stimulus for remodeling; but that additional work is warranted to confirm that the stimulus from sprint exercise is insufficient.

Another novel finding of this study is that arterial diameter responses evoked during sprint exercise differ from moderate intensity (Figure 4, Table 4). To our knowledge, these responses have not been previously reported. We speculated that greater vasoconstriction could differentially affect shear patterns, primarily by increasing retrograde shear rate. Interestingly, the time course of brachial artery diameter change did not align with that of increasing retrograde shear rates following the second sprint, suggesting that other factors were involved. Brachial diameter decreased early on after the first sprint (S1M3) and did not further decrease throughout the bout in a similar manner with the retrograde shear (Figure 4). Furthermore, retrograde shear remained elevated at 10‐ and 15‐min post exercise as brachial artery diameter returned toward baseline values. Given this mismatch in time course, we find it unlikely that large conduit artery vasoconstriction played a role. One possibility is that vasoconstrictor responses in downstream resistance vessels in the arm moderated retrograde shear, although no measures were taken to assess this (e.g., forearm vascular conductance). Previous work suggests, however, that cutaneous forearm vascular conductance may be a key determinant of brachial artery retrograde shear during continuous cycling exercise (Simmons et al., 2011). Future research incorporating such measures during intense intermittent exercise could add to the body of literature investigating cardiovascular control.

## 5 LIMITATIONS

### 5.1 Generalizability

Participants were young and healthy, and these results may differ with advancing age or disease states that affect cardiovascular or metabolic control. For example, recent work suggests that exercise pressor and metaboreflex responses are exaggerated in even relatively young individuals with metabolic syndrome (Stavres et al., 2023). These differences could impact vasoconstrictor responses and thus flow/shear patterns. Future research is needed to investigate such effects.

### 5.2 Measurement specificity

The data are limited to measures in the brachial artery. Incorporating femoral artery blood flow measurements would yield valuable insight into cardiovascular control, helping to determine the redirection of blood flow to the working limbs, as well as the potential for lasting effects in the lower body. Some other sites that could indicate systemic shear (e.g., carotid artery) may also corroborate findings in the arm, providing a more robust representation of systemic shear.

### 5.3 Doppler ultrasound

Lastly, ultrasound measurements are limited to large conduit arteries in general, and some measure of vascular conductance in smaller resistance vessels would provide valuable mechanistic insight into what may drive differing shear patterns. The ultrasound measurements are indicative of limb blood flow; though it is challenging to ascertain whether this flow is directed to skeletal muscle or cutaneous (micro) vascular beds (Limberg et al., 2019). While also subject to limitations, complementary use of near‐infrared spectroscopy‐based technologies or laser Doppler flowmetry may also be informative. Though not part of the study aims, these additions could help contextualize findings further in future work.

## 6 CONCLUSION

The results from this study suggest that there are lasting effects of sprint exercise on shear patterns in non‐local vasculature. With repeated exposure through regular exercise training, these effects could be beneficial for maintaining endothelial NO bioavailability and could promote vascular endothelial remodeling. These findings are important in the context of using brief exercise bouts to attenuate the negative impact of prolonged inactivity. Future exercise training studies should continue investigating the impact of these modalities on vascular function outcomes.

## AUTHOR CONTRIBUTIONS

All authors made substantial contributions to the study and are accountable for the work outlined in this manuscript. MC and MJ were responsible for the original conceptualization and methodology of the study, with further help from LK. MC, LK, and MJ were responsible for project administration and investigation. MC was responsible for formal analysis, visualization, and writing the original draft of the manuscript. LK and MJ were responsible for reviewing and editing the manuscript. Specific author contributions were based on Contributor Roles Taxonomy (CRediT) that were most applicable to the study.

## FUNDING INFORMATION

No funding information provided.

## CONFLICT OF INTEREST STATEMENT

The authors report there are no competing interests to declare.

## ETHICS STATEMENT

The study protocol received ethical approval by the University of Mississippi Institutional Review Board (22‐042 & 23‐017) and was conducted in line with the ethical principles of the Declaration of Helsinki. Participants gave written consent to take part in the study after being informed of all procedures, risks, and study objectives.

## Data Availability

The data that support the findings of this study can be made available from the corresponding author upon reasonable request.
